# Hypoglycemic Encephalopathy Caused by Insulinoma With Reversible High Signals in the White Matter on Diffusion‐Weighted MRI: A Case Report

**DOI:** 10.1155/crra/7322838

**Published:** 2026-06-12

**Authors:** Taira Shiratori, Iichiro Osawa, Wataru Watanabe, Keita Nagawa, Koichiro Matsuura, Shinji Kakemoto, Morihiro Higashi, Eito Kozawa

**Affiliations:** ^1^ Department of Radiology, Saitama Medical University Hospital, Saitama, Japan, saitama-med.ac.jp; ^2^ Department of Radiology, Saitama Medical Center, Saitama Medical University, Saitama, Japan, saitama-med.ac.jp; ^3^ Department of Pathology, Saitama Medical Center, Saitama Medical University, Saitama, Japan, saitama-med.ac.jp

**Keywords:** case report, diffusion-weighted imaging, hypoglycemic encephalopathy, insulinoma, white matter

## Abstract

Hypoglycemic encephalopathy can result in reversible, and sometimes irreversible, lesions within the brain that need prompt recognition and treatment. The disease is relatively uncommon, but radiologists must keep in mind the rare radiologic findings of this disease. Although the most common cause is insulin, insulinoma is a rare etiology, and hypoglycemia is often misdiagnosed as epilepsy. A 66‐year‐old man presented with recurrent involuntary movements and impaired consciousness with neuropsychiatric symptoms without a history of antidiabetic therapy. Initial blood glucose was critically low at 29 mg/dL. Diffusion‐weighted imaging (DWI) revealed reversible high signals in the white matter, involving the bilateral posterior limbs of the internal capsule, corona radiata, cerebral peduncle, and splenium of the corpus callosum. A fasting test and contrast‐enhanced computed tomography confirmed the diagnosis of insulinoma. Intravenous glucose was administered immediately, resulting in rapid clinical improvement. Laparoscopic distal pancreatectomy was subsequently performed. Clinical symptoms resolved completely within 1 day of glucose administration. Follow‐up DWI performed 2 months after onset demonstrated complete resolution of all white matter hyperintensities without recurrence. The patient reported feeling well without recurrence of the symptoms. We report a case of hypoglycemic encephalopathy caused by insulinoma with reversible high signals in the white matter on DWI, presenting with neuropsychiatric symptoms mimicking epilepsy. We also discuss findings of magnetic resonance imaging in hypoglycemic encephalopathy with emphasis on the time course of the findings.

## 1. Introduction

Hypoglycemia causes neurological symptoms ranging from weakness and confusion to seizures and coma, and can result in both reversible and irreversible brain lesions on magnetic resonance (MR) imaging (MRI). Although lesions typically involve the cortex, basal ganglia, and hippocampus [1], DWI may reveal a distinct white matter pattern, including the splenium of corpus callosum (SCC), posterior limb of internal capsule (PLIC), corona radiata, centrum semiovale, and cerebral peduncle [2–5].

Hypoglycemic encephalopathy presents important diagnostic pitfalls for radiologists. DWI abnormalities overlap with a broad differential diagnosis and may be misattributed to other conditions when the clinical context is incomplete. Furthermore, when hypoglycemia presents exclusively with neuroglycopenic symptoms without autonomic warning signs, it is frequently misdiagnosed as epilepsy, and this diagnostic delay can result in vegetative state or death. Hypoglycemia results most commonly from overuse of insulin, whereas insulinoma is a rare etiology that is particularly prone to these pitfalls. In addition, sequential MRI findings in hypoglycemic encephalopathy have been poorly discussed in the current literature.

Here we report a case of insulinoma‐induced hypoglycemic encephalopathy presenting with neuroglycopenic symptoms mimicking epilepsy, with three contributions to the literature: (1) It illustrates the diagnostic pitfalls described above in a single patient; (2) it demonstrates reversible high signals in the SCC and bilateral pyramidal tracts on DWI, highlighting the characteristic white matter pattern that should alert radiologists to this diagnosis; and (3) it documents complete radiological reversibility with serial imaging, supporting a conceptual framework of serial MRI findings that provides a reference for the expected time course of lesion evolution and normalization.

## 2. Case Presentation

A 66‐year‐old man presented with an attack of involuntary movement in the right arm and speech disturbance approximately 1 year before admission. There was no past history of diabetes mellitus. He did not use oral hypoglycemic agents such as sulfonylureas or insulin. There was no history of alcohol abuse or nutritional disorder. He did not use illicit drugs. There was no family history of endocrine disorder or epilepsy. The patient had no other symptoms associated with hypoglycemia or insulinoma such as palpitations, tremors, sweating, hyperphagia, or weight gain, and blood glucose levels were not assessed. He was diagnosed with temporal lobe epilepsy and started on antiepileptic medication (carbamazepine and gabapentin). Despite escalation of the treatment, symptoms worsened gradually. On the day of admission, he moved his limbs randomly and made abnormal vocalizations, and was transferred to authors′ hospital. The clinical timeline is summarized in Table 1.

**Table 1:** Timeline of clinical course.

Time point	Category	Clinical events/findings
~1 year before admission	Symptom onset and prior treatment	Onset of recurrent involuntary movements of the right arm and speech disturbance. Blood glucose not assessed. Misdiagnosed as temporal lobe epilepsy; antiepileptic therapy initiated (carbamazepine and gabapentin). Symptoms worsened gradually despite treatment escalation.
Day of admission	Admission	Acute deterioration with random limb movements and abnormal vocalization. Transferred to authors′ hospital. On admission: unconscious, sedated with haloperidol and diazepam. Initial venous blood glucose: 29 mg/dL (not immediately recognized).
17.5 h after admission	Imaging	Brain CT: no abnormality detected.
18 h after admission	Imaging	Initial brain MRI including DWI: hyperintensities in bilateral entire PLIC, corona radiata, cerebral peduncle, and SCC with reduced ADC values. T1WI, T2WI, FLAIR, and SWI: no abnormalities. Lesion distribution did not correspond to a specific vascular territory. MR angiography: no stenosis.
~18.5 h after admission (30 min after MRI)	Diagnosis and treatment	Venous blood glucose confirmed at 20 mg/dL. Immediate IV injection of 40 mL of 50% glucose solution. Capillary blood glucose subsequently measured at 67 mg/dL. Consciousness improved; random limb movements completely disappeared.
Within 1 day after admission	Clinical course	Continuous IV glucose infusion started. All symptoms gradually returned to normal within 1 day.
5 days after admission	Endocrine workup and imaging	Fasting test: glucose 33 mg/dL (at 2 h), insulin 24 *μ*U/mL (ref. 1.1–17), C‐peptide 5.39 ng/mL (ref. 0.61–2.09). All insulinoma indices positive (Fajans 0.73, Grunt 1.38, Turner 8000). Anti‐insulin antibody 0.4%. Contrast‐enhanced CT of the abdomen: well‐defined arterially enhancing mass, 20 mm in diameter, in the pancreatic tail—consistent with insulinoma.
19 days after admission	Surgery	Laparoscopic distal pancreatic resection performed. No postoperative complications. Blood glucose levels returned to normal. Histopathology confirmed insulinoma (G1 neuroendocrine tumor, Ki‐67 < 2%, WHO 2022 classification).
2 months after admission	Follow‐up	Follow‐up DWI: complete resolution of all white matter hyperintensities in bilateral cerebral peduncle, SCC, PLIC, and corona radiata. Patient reported feeling well without recurrence of symptoms.

Abbreviations: ADC, apparent diffusion coefficient; CT, computed tomography; DWI, diffusion‐weighted imaging; FLAIR, fluid‐attenuated inversion recovery, IV, intravenous; MRI, magnetic resonance imaging; PLIC, posterior limb of internal capsule; SCC, splenium of corpus callosum; SWI, susceptibility‐weighted imaging; T1WI, T1‐weighted imaging; T2WI, T2‐weighted imaging.

**Figure 1:**
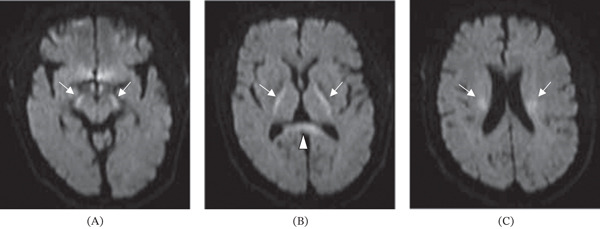
Magnetic resonance imaging of hypoglycemic encephalopathy. Axial diffusion‐weighted imaging performed 18 h after admission depicts hyperintensities of (A, arrows) bilateral cerebral peduncle, (B, arrowhead) splenium of corpus callosum, (B, arrows) posterior limb of internal capsule, and (C, arrows) corona radiata.

At admission, he was unconscious, sedated with haloperidol and diazepam. Though he was awake without stimuli, he was unable to recall his name and date of birth. The systolic blood pressure was 170 mmHg, and the oxygen saturation was 99% under administration of 3 L/min of oxygen through a facial mask. The pupils were 2 mm in diameter and reacted to light. Levels of electrolytes, calcium, total protein, albumin, amylase, lipase, and C‐reactive protein were normal, as were the results of tests of the liver and renal function. The white‐cell count was 9800/mm^3^, but the remainder of the hematologic profile was within reference ranges. Initial venous blood glucose was 29 mg/dL measured in the emergency department, which was not noticed until initial MRI was performed. Computed tomography (CT) of the brain performed 17.5 h after admission was normal. Subsequently, initial DWI performed 18 h after admission disclosed hyperintensities in the bilateral entire PLIC, corona radiata, cerebral peduncle, and SCC (Figure 1) with reduced apparent diffusion coefficient values. T1‐, T2‐, and susceptibility‐weighted and fluid‐attenuated inversion recovery images showed no abnormalities. Symmetrical involvement of SCC was noted with extension from the central to lateral portions. The topographic features of the lesion did not correspond to a specific vascular territory. MR angiographic examination of the head disclosed no stenosis. His venous blood glucose level was 20 mg/dL at 30 min after the first MRI examination, and 40 mL of 50% glucose solution was injected immediately. Subsequently, the capillary blood glucose level was measured as 67 mg/dL, his consciousness level improved, and random limb movements completely disappeared.

**Figure 2:**
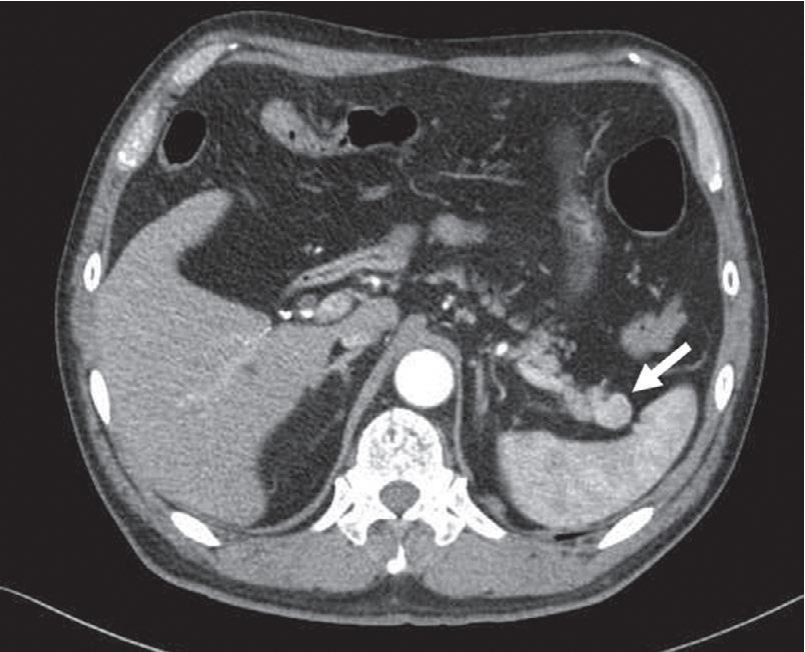
Computed tomography of insulinoma. Arterial‐phase pancreatic computed tomography shows a homogeneously enhancing mass, 20 mm in diameter (arrow), in the tail of the pancreas. On the portal venous phase, there was persistent enhancement as compared with the pancreatic parenchyma (not shown).

**Figure 3:**
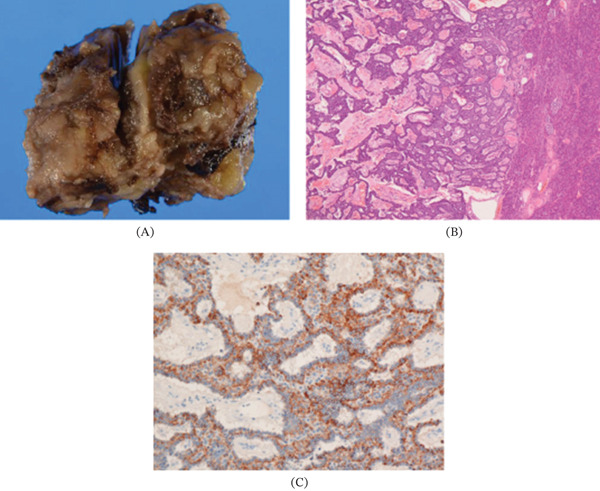
Pathology of insulinoma. A gross photograph of the resected pancreas shows a mass of (A) 30 × 22 × 20 mm. Microscopic examination of a section of the (B) pancreatic mass reveals a well‐circumscribed neoplasm, and immunohistochemical staining of the tumor cells is positive for chromogranin A, synaptophysin, CD56, and (C) insulin. The tumor shows a Ki‐67 index below 2% and no mitotic figures, consistent with a well‐differentiated (G1) neuroendocrine tumor under the 2022 WHO classification.

**Figure 4:**
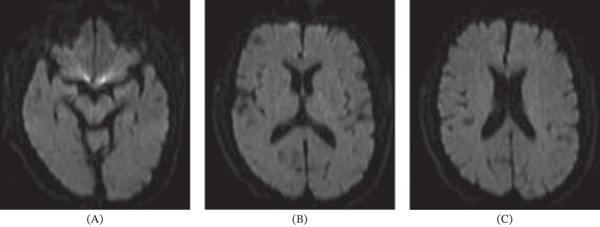
Follow‐up magnetic resonance imaging of hypoglycemic encephalopathy. Axial diffusion‐weighted imaging performed 2 months after admission shows complete resolution of hyperintensities in (A) bilateral cerebral peduncle, (B) splenium of corpus callosum, (B) posterior limb of internal capsule, and (C) corona radiata.

After continuous intravenous glucose infusion was started, his symptoms had gradually returned to normal within 1 day. Critical samples were not collected at the time of hypoglycemia, whereas a fasting test was performed 5 days after admission. The glucose level 2 h after fasting was 33 mg/dL, with a concomitant insulin level of 24 *μ*U/mL (reference range, 1.1–17 *μ*U/mL) and C‐peptide level of 5.39 ng/mL (reference range, 0.61–2.09 ng/mL). The Fajans, Grunt, and Turner indices, diagnostic indicators of insulinoma, were all positive (Fajans 0.73 [positive, > 0.3], Grunt 1.38 [positive, < 2.5], Turner 8000 [positive, > 200]). The anti‐insulin antibody‐binding rate was 0.4% (reference range, 0.00%–0.30%). Contrast‐enhanced CT of the abdomen performed 5 days after admission showed a well‐defined and enhancing mass on the arterial phase, 20 mm in diameter, in the tail of the pancreas (Figure 2). These findings made a diagnosis of insulinoma most probable. The patient underwent laparoscopic distal pancreatic resection 19 days after admission. Histological examination of the pancreatic tumor confirmed the diagnosis of insulinoma (Figure 3). Postoperatively, no surgical complication occurred and glucose levels returned to normal. The patient tolerated all interventions without complication. Adherence to follow‐up was maintained, with repeat DWI performed 2 months after onset confirming complete resolution of hypersignals observed on initial DWI (Figure 4). He reported feeling well without recurrence of the symptoms.

## 3. Discussion

The DWI distribution pattern of hypoglycemic encephalopathy is characteristic and classified into white and gray matter lesions [3]. White matter involvement is variable with a focal or diffuse distribution, affecting the SCC, PLIC, corona radiata, centrum semiovale, and cerebral peduncle, whereas gray matter lesions are divided into cortical and basal ganglia lesions. Splenial lesions are mainly divided into two types based on shape and extent. The first type shows wide involvement with poorly defined borders [6–8], typical of hypoglycemia as in the present case. The second type presents as ovoid to round lesions with sharp borders in the mid‐portion of the splenium, as seen in clinically mild encephalitis/encephalopathy with a reversible splenial lesion (MERS) and splenial lesions associated with antiepileptic drugs [9–12]. PLIC involvement is classified into two types: focal, with a round lesion usually in the posterior one third [2, 6, 13], suggestive of the corticospinal tract and diffuse, with a linear pattern throughout the entire PLIC [14], as in our case. On the other hand, the brain stem, cerebellum, and thalamus are often unaffected on MRI [1].

The temporal evolution of MRI findings in hypoglycemic encephalopathy is variable [6, 15, 16] and has been poorly discussed. The timing of MRI relative to glucose administration is crucial, as lesions reverse after glucose normalization. During the early acute phase, focal white matter alterations predominate on DWI [2, 8, 17, 18], whereas in the late acute phase, diffuse white matter and gray matter involvement may appear [17, 19, 20]. Lesions did not appear on T2‐weighted imaging until at least 12 h after symptom onset [21]. The SCC and/or PLIC may be affected earlier than other white matter structures [2, 17, 18], with DWI high signals reported as early as 45 min after symptom onset [21], whereas hyperacute hypoglycemia of less than 10 min showed no changes [22]. Splenial and PLIC alterations almost completely recover within as early as 2 h after glucose infusion [13]. PLIC involvement can extend into the pyramidal tracts such as the corona radiata, but not into the brain stem [3, 16], consistent with its resistance to hypoglycemia. Lee et al. [15] showed the cortex was involved after white matter involvement disappeared. Of the gray matter structures, the basal ganglia are less frequently affected in isolation than the cortex [1, 3, 7], and both are involved coincidentally [1, 3, 7], suggesting cortical involvement precedes basal ganglia involvement.

Reversibility of hypoglycemic encephalopathy encompasses both clinical and radiological aspects, and both reversible and irreversible forms have been reported, including in insulinoma‐induced cases [6, 23]. Although some reports of insulinoma‐induced hypoglycemic encephalopathy describe irreversible outcomes [6, 23], our case uniquely demonstrates complete recovery in both clinical and radiological terms. Although “radiological reversibility” is ambiguous, here we define it as complete recovery of all MRI sequences at any speed including morphological changes, and limit the following discussion to radiological reversibility. Reversibility may not be related to the speed of recovery. More focal white matter involvement is more reversible, whereas diffuse white matter and/or gray matter involvement is usually irreversible [15, 19]; therefore, reversibility may be related to lesion distribution. Brain lesions of hypoglycemia are characterized by rapid change after glucose administration [13, 24], with almost complete recovery occurring within as early as 2 h after glucose infusion [13], a time course shorter than that of other reversible brain lesions [10–12, 25, 26]. Table 2 presents a conceptual framework of serial MRI findings in hypoglycemic encephalopathy, including lesion appearance, reversibility, and outcome, synthesized from the published literature to provide a structured reference for radiologists interpreting DWI in this clinical setting.

**Table 2:** Conceptual framework of serial MRI findings in hypoglycemic encephalopathy: proposed order of lesion appearance, reversibility, and associated outcome, synthesized from the published literature. The findings of the present case are consistent with this framework.

	Brain lesions		Gray matter	
	White matter			
Order of appearance on DWI	Focal (SCC/PLIC, followed by other white matter)	Diffuse	Cortex	Basal ganglia
Reversibility	Reversible	Irreversible	Irreversible	Irreversible
Outcome	Good	Poor	Poor	Poor

Abbreviations: DWI, diffusion‐weighted imaging; MRI, magnetic resonance imaging; PLIC, posterior limb of internal capsule; SCC, splenium of corpus callosum.

Although the imaging‐focused differential diagnosis of isolated transient splenial lesions is broad [27], simultaneous involvement of the splenium and pyramidal tracts is rarely encountered and narrows the differential considerably. The simultaneous predilection may be explained by their higher myelin water content compared with other white matter structures in normal individuals [28]. On imaging, Marchiafava–Bignami disease and other encephalitis/encephalopathy also involve the SCC and other white matter reversibly, but may recover without treatment or disappear gradually [25, 29, 30]. No history of alcohol abuse in our case also argues against Marchiafava–Bignami disease. Splenial lesions associated with antiepileptic drugs, such as carbamazepine and gabapentin, are typically isolated, well‐circumscribed, and oval‐shaped on DWI, occurring after drug withdrawal [11, 12, 31]. In contrast, SCC involvement in hypoglycemia extends to the lateral splenium with ill‐defined borders, sometimes including extracallosal white matter changes, and in our case DWI abnormalities persisted despite no drug withdrawal. Therefore, concurrent of SCC and pyramidal tract involvement with rapid recovery after glucose administration may be a characteristic imaging finding of hypoglycemia. Acute ischemic stroke is the most urgent mimic. However, hypoglycemic lesions do not conform to a vascular territory and are typically bilateral and symmetric. MR angiography, which was normal in our case, helps exclude large‐vessel occlusion.

Insulinoma causes hypersecretion of insulin and results in hypoglycemia. Although severe hypoglycemia is a known trigger for seizures, it is frequently confounded with primary epilepsy. Establishing a causal chain from seizure to hypoglycemia, and finally to insulinoma, is often difficult, and this complexity can lead to a diagnostic delay, as seen in our case. As the majority of insulinomas are curable by surgical operation, early differentiation from epilepsy is of critical importance. In our case, the patient presented exclusively with neuroglycopenic symptoms, notably lacking autonomic signs such as palpitations, tremors, or sweating. This absence of autonomic warning signals may be attributed to hypoglycemia‐associated autonomic failure (HAAF), in which recurrent hypoglycemic episodes blunt the sympathoadrenal response and shift the symptomatic threshold, potentially masking the early clinical manifestations of hypoglycemia.

Our study has several limitations. A standardized endocrine workup including critical sample collection during the acute phase was not fully performed due to the patient′s critical condition, the emergency setting, and a lack of initial clinical suspicion for hypoglycemia. Although the diagnosis of insulinoma was subsequently confirmed by imaging, fasting test, and histopathology, the lack of real‐time biochemical validation remains a limitation.

In conclusion, hypoglycemic encephalopathy due to insulinoma should be suspected when seizure‐like episodes occur in patients without antidiabetic therapy, particularly when characteristic white matter DWI findings are present. Although our diagnosis and treatment were not based on an established protocol for hypoglycemia/insulinoma, these imaging findings should prompt immediate serum glucose measurement and a search for the underlying etiology to facilitate timely diagnosis and treatment.

## Funding

No funding was received for this manuscript.

## Ethics Statement

All procedures performed in studies involving human participants were in accordance with the ethical standards of the institutional and/or national research committee and with the 1964 Helsinki declaration and its later amendments or comparable ethical standards. This case report was written in accordance with the 2013 CARE (CAse REport) guidelines.

## Consent

Written informed consent for publication was waived because the patient was lost to follow‐up after completion of treatment and subsequent outpatient care. Institutional review board approval was not required for a single case report in accordance with local policy. In accordance with the journal′s policy and ICMJE guidelines, the patient cannot be identified from the clinical and imaging information provided, and the benefits of publication outweigh any residual risk to patient privacy. No personally identifiable information, including name, initials, date of birth, or exact dates of hospitalization, has been included in the manuscript or figures.

## Conflicts of Interest

The authors declare no conflicts of interest.

## Data Availability

Imaging and clinical data may be obtained by contacting the corresponding author and via the Department of Radiology at Saitama Medical University Hospital.
